# Errata

**Published:** 2013-09-06

## Vol. 62, No. RR-03

In the Recommendations and Reports, “Diagnosis and Management of Q Fever — United States, 2013: Recommendations from CDC and the Q Fever Working Group” on page 6, in Table 2, the following errors occurred.

In the third column, “Children,” the recommended treatment of acute Q fever for children aged <8 years with mild or uncomplicated illness, should read as follows: “Doxycycline 2.2 mg/kg per dose twice a day for 5 days (maximum 100 mg per dose). If patient remains febrile past 5 days of treatment: trimethoprim/sulfamethoxazole 4–20 mg/kg**/24 hours (dose based on trimethoprim component) in equally divided doses every 12 hours (maximum: 320 mg trimethoprim per 24 hours)**”

In the fourth column, “Pregnant women,” the recommended treatment of acute Q fever for pregnant women should read as follows: “Trimethoprim/sulfamethoxazole: 160 mg/800 mg twice a day throughout pregnancy **but not beyond 32 weeks’ gestation**^††^

In the ^¶^ footnote, the following sentence should be added at the end: “**Trimethoprim/sulfamethoxazole is contraindicated in children aged <2 months.**”

In the ^††^ footnote, the following sentence should be added at the end: “**Trimethoprim/sulfamethoxazole should be discontinued for the final 8 weeks of pregnancy because of the risk for hyperbilirubinemia**.”

